# On the traces of *tcf12*: Investigation of the gene expression pattern during development and cranial suture patterning in zebrafish (*Danio rerio*)

**DOI:** 10.1371/journal.pone.0218286

**Published:** 2019-06-12

**Authors:** Rabea Blümel, Miriam Zink, Eva Klopocki, Daniel Liedtke

**Affiliations:** 1 Institute of Human Genetics, Biocenter, Julius-Maximilians-University Würzburg, Würzburg, Germany; 2 Comprehensive Heart Failure Center and Department of Internal Medicine I, University Hospital Würzburg, Würzburg, Germany; University of Texas Southwestern Medical Center, UNITED STATES

## Abstract

The *transcription factor 12* (*tcf12*) is a basic Helix-Loop-Helix protein (bHLH) of the E-protein family, proven to play an important role in developmental processes like neurogenesis, mesoderm formation, and cranial vault development. In humans, mutations in *TCF12* lead to craniosynostosis, a congenital birth disorder characterized by the premature fusion of one or several of the cranial sutures. Current research has been primarily focused on functional studies of *TCF12*, hence the cellular expression profile of this gene during embryonic development and early stages of ossification remains poorly understood. Here we present the establishment and detailed analysis of two transgenic *tcf12*:*EGFP* fluorescent zebrafish (*Danio rerio*) reporter lines. Using these transgenic lines, we analyzed the general spatiotemporal expression pattern of *tcf12* during different developmental stages and put emphasis on skeletal development and cranial suture patterning. We identified robust *tcf12* promoter-driven EGFP expression in the central nervous system (CNS), the heart, the pronephros, and the somites of zebrafish embryos. Additionally, expression was observed inside the muscles and bones of the viscerocranium in juvenile and adult fish. During cranial vault development, the transgenic fish show a high amount of *tcf12* expressing cells at the growth fronts of the ossifying frontal and parietal bones and inside the emerging cranial sutures. Subsequently, we tested the transcriptional activity of three evolutionary conserved non-coding elements (CNEs) located in the *tcf12* locus by transient transgenic assays and compared their *in vivo* activity to the expression pattern determined in the transgenic *tcf12*:*EGFP* lines. We could validate two of them as *tcf12* enhancer elements driving specific gene expression in the CNS during embryogenesis. Our newly established transgenic lines enhance the understanding of *tcf12* gene regulation and open up the possibilities for further functional investigation of these novel *tcf12* enhancer elements in zebrafish.

## Introduction

TCF12, also called HEB or HTF4, is a member of the bHLH protein family, widely expressed in many vertebrate tissues and cell lines. TCF12 can form homodimers and heterodimers with other bHLH proteins to regulate the transcription of various target genes during different developmental processes, like mesodermal and hematopoietic specification as well as T cell development [1–3]. In rodent models, *Tcf12* has been shown to be involved in neurogenesis and loss of *Tcf12* leads to a reduction in brain size [4–6]. Recent studies assigned an essential role for *Tcf12* in osteogenic differentiation of bone marrow stem cells via BMP and Erk1/2 signaling pathways [7]. A number of mutations affecting *TCF12* have been identified in patients with coronal craniosynostosis type 3 (OMIM: #615314), prompting that *TCF12* plays a key role in coronal suture development and patency [8, 9]. Cranial sutures are bands of non-ossified mesenchymal tissue that separate the calvarial bone plates during vertebrate skull development. They are characterized as highly proliferative zones of osteogenic differentiation and bone formation. Under normal conditions, only the metopic suture fuses during early childhood in humans whereas the other sutures are open up to an age of 40 years [10]. The patency of the sutures during childhood is essential to enable normal skull growth in compliance with the developing brain. Depending on the affected suture that is fused, craniosynostosis patients can exhibit severe skull malformations, compensatory bone growth, facial asymmetries, and raised intracranial pressure [11]. Most cases of craniosynostosis are treated by surgery of affected newborns at an age between 8 and 15 months to reduce intracranial pressure and for correction of the cranial deformity [12]. A cure or even a pharmacological treatment is not available, as the molecular reasons for the suture fusions are heterogeneous and often still unknown [13].

To gain a deeper understanding of the complex functions of disease-relevant genes, like the transcription factor *tcf12* during cranial vault development, the generation of an appropriate animal model is eligible. Besides mice, rats, and rabbits, over the last decade zebrafish have been successfully used as a vertebrate organism to model different bone and skull disorders including craniosynostosis [14–16]. Obviously, skull shape and size are very different between human and zebrafish, yet the anatomy of the skull vault and the interposed sutures show a striking similarity to that of mammals [14, 17]. Moreover, essential signaling pathways and a large number of cellular processes during skull development are conserved between species and allow identification of general developmental processes [14, 15, 18]. In contrast to other vertebrate model organisms, zebrafish have a short generation time, transparent embryos and show a rapid *ex utero* development allowing visual *in vivo* analysis of craniofacial elements during development. Non-invasive methods for visualization of growth processes during skull development are well established in this species and enable *in vivo* investigation over time at cellular resolution [19, 20].

Comparative studies in mice and zebrafish showed, that loss of *Tcf12* together with the loss of its interaction partner *Twist1* results in fusions of the coronal sutures in both species, whereas mutations in *Tcf12* alone do not lead to suture fusions [9, 21]. Although different functional studies have contributed significantly to unravel the molecular role of *TCF12* in cranial suture patterning, the spatiotemporal expression pattern of *TCF12* during ossification of the skull bones and during suture patency remain poorly described. In this study, we established two *Tg(tcf12*:*EGFP)* fluorescent reporter lines to determine the expression pattern of *tcf12* in zebrafish embryos, juvenile and adult fish in detail. In addition to the investigation of the general expression pattern in multiple tissues, we placed a special focus on investigating the cellular expression pattern of *tcf12* during cranial and suture patterning.

The broad *TCF12* expression pattern and its very specific effect on cranial sutures after mutation imply an expressional regulation by a number of different upstream factors. *Cis*-acting enhancer elements within the human *TCF12* locus have been reported and investigated in transgenic mouse models [22]. These experiments hint to a potential tissue specific regulation of *TCF12* by these elements during development and foreshadow regions harboring essential transcription factor binding sites. Zebrafish are known to be a valuable model for identifying and validating such conserved noncoding elements (CNEs) [23–25]. The advantage of taking zebrafish as a model for CNE studies lies in the long evolutionary divergence time that exists between humans and zebrafish since their last common vertebrate ancestor of approx. 450 Mya. Human-rodent comparisons, by contrast, are limited due to a comparatively short evolutionary divergence time which comes along with a high overall similarity even in nonfunctional genomic regions [22]. To validate such regulatory elements in zebrafish we compared the *tcf12* expression pattern characterized by our newly established transgenic lines to the transcriptional activity of three different *TCF12* CNEs *in vivo*. To investigate the CNE activity during embryogenesis we used the ZED vector, in which CNE sequences combined with a minimal promoter drive fluorescence reporter genes [26].

## Material and methods

### Animal maintenance

Laboratory zebrafish embryos of the *mitfa*^*w2/w2*^; *mpv17*^*b18/b18*^ strains (ZFIN ID: ZDB-GENO-121010-3) were raised as described by Westerfield [27] under standard laboratory conditions at 28.5° C. Embryos were staged by morphological characteristics according to Kimmel et al. and Parichy et al. [28, 29]. All procedures involving experimental animals were performed in compliance with German animal welfare laws, guidelines, and policies. The protocol was approved by the Committee on the Ethics of Animal Experiments of the University of Würzburg and the ‘*Regierung von Unterfranken*’ (Permit Number: 55.2 2532-2-428).

### Cloning of *tcf12*:*EGFP* promoter constructs

For generating transgenic *tcf12*:*EGFP* zebrafish, the Tol2 transposon system was used [30–32]. To build *tcf12*:*EGFP* promoter constructs, the sequence upstream of the transcriptional start site of *tcf12* (*tcf12*-201 ENSDART00000009938.11) was amplified from zebrafish genomic DNA using Q5 High-Fidelity DNA Polymerase (New England Biolabs, Ipswich, MA, USA). For the PCR we used specific primers containing *att*B4 and *att*B1R Gateway recombination sites (sequences in S1 Table). The amplified fragment was cloned into a Gateway pDONR P4-P1R entry vector using Gateway BP Clonase II enzyme mix (Invitrogen/Thermo Scientific, Waltham, MA). By restriction digestion, two different constructs were subsequently investigated: one which contains a 232 bp minimal *tcf12* promoter fragment and one containing a 2158 bp promoter fragment. Both fragments target the *tcf12* reference transcripts tcf12-210 (ENSDART00000174218.2) and tcf12-201 (ENSDART00000009938.11), validated by the Ensembl and manually annotated HAVANA databases and also the transcripts tcf12-211 (ENSDART00000174292.2) and tcf12-212 (ENSDART00000174335.2; see S2 Table for an overview of all transcripts). The plasmids were recombined into the destination vector pTolDestR4R2pA together with a second entry vector pENTR-EGFP using the LR Clonase II enzyme mix (Invitrogen/Thermo Scientific, Waltham, MA), resulting in the corresponding *tcf12*:*EGFP* plasmids. Plasmids were kindly provided by the Nathan D. Lawson lab, University of Massachusetts or are parts of the Tol2kit (Addgene) [33].

### Microinjection and transgenic line establishment

One cell stage zebrafish embryos were injected with solutions comprising of *tcf12*:*EGFP* plasmids [25 ng/μl each] along with Tol2 transposase mRNA [50 ng/μl] and phenol red (pH 7.0; 0.05% final concentration; for visualization of injection solution). Tol2 transposase-encoding mRNA was *in vitro* transcribed from NotI-linearised pCS-TP vector [31] via SP6 RNA polymerase using the mMESSAGE mMACHINE kit (Ambion/ Life Technologies, Darmstadt, Germany). Positive injected embryos were identified 24 and 48 hours post-fertilization (hpf) by transient green fluorescence and were raised for further analyses and transgenic line establishment.

### Whole mount RNA *in-situ* hybridization

Whole mount RNA *in-situ* hybridization was performed according to standard protocols [34]. RNA probes were synthesized from cloned partial mRNA sequences of *tcf12* (ENSDART00000009938.11) amplified from embryonic zebrafish cDNAs using the DIG RNA Labeling Kit (Roche, Basel, Switzerland, primer sequences in S1 Table). Sense probes were synthesized and served as negative control for each anti-sense probe. The cloned probe targets a common sequence at the 5´end and UTR region which is shared by different zebrafish *tcf12* transcripts and therefore detects several mRNA transcripts (see S2 Table for an overview of all targeted *tcf12* transcripts).

### Bone staining protocols and imaging

Visualization of bone structure was performed by incubation in alizarin red solution (50 μg/ml alizarin red diluted in aquarium fish water, 10 mM HEPES; pH 7.0) for 2 d and washing twice for 1h in fish water before mounting and imaging. Imaging was performed either with a Zeiss SteREO Lumar stereomicroscope or a Nikon A1+ confocal microscope. Alizarin red fluorescence was imaged by using the excitation and emission spectra for DsRed (excitation: 558 nm; emission: 583 nm). Subsequent quantifications and image processing were done by ImageJ (version 1.50g; Fiji bundle; https://fiji.sc/) and CorelDraw X6 Graphics Suite (Corel Corporation).

### Immunohistochemical (IHC) staining of cryosections

For IHC staining adult *Tg(-2*.*1tcf12*:*EGFP)* fish were euthanized by Tricaine incubation according to Matthews [35] and fixed in 4% PFA/PBST (Paraformaldehyde; Phosphate buffered saline with 0.5% Tween 20) at 4°C overnight. Subsequently, the heads of the fish were disembodied, dehydrated in a Methanol/PBST dilution series and stored in 100% Methanol at −20°C. For cryoprotection, the heads were rehydrated in a reverse Methanol dilution series and incubated in 30% sucrose at 4°C overnight. The cryoprotected heads were embedded in OCT medium (Sakura Finetek, USA) and sectioned in 6 μm thick slices. Cryosections were transferred onto Superfrost plus slides (Thermo Scientific) and stored at -20°C. For EGFP detection the slides were washed in Tris-HCl buffer (pH 7.6) 3 x 5 min and then incubated in blocking buffer (Tris-HCl at pH 7.6 with 0.05% Tween 20 and 0.1% bovine serum albumin) for 30 min. After blocking, the sections were immunostained for 1.5 h with a chicken anti-GFP antibody (Abcam, ab13970; Antibody Registry No.: AB_300798) diluted 1:300 in blocking buffer. After washing, the slides were incubated in the dark for 1h in secondary Alexa Fluor antibody (Abcam, ab175477) diluted 1:1000 in the same buffer. For Phalloidin staining, slides were washed in permeabilization buffer (0.5% Triton X-100 in PBS) for 3x 5 min and then stained in Acti-stain™ 488 Phalloidin (Cytoskeleton, Inc.) diluted 1:100 and DAPI diluted 1:1.000 in PBST for 30 min. After washing for 3 x 5 min in PBST, slides were embedded and imaged on a Nikon A1+ confocal microscope.

### Functional enhancer analyses in zebrafish

To test the regulatory ability of three conserved *tcf12*-CNEs in zebrafish, we sub-cloned all three sequences separately in the Gateway donor vector pDONR221 (Thermo Scientific, Waltham, MA). Subsequently, we recombined the CNEs into the zebrafish Enhancer Detector (ZED) vector, which contains Tol2 sites, a minimal promoter linked to GFP and a *cardiac-actin*:RFP cassette that serves as control for transgenesis efficiency. Transient transgenesis was performed as previously described [26]. We analyzed at least three independently injected pools of transient transgenic embryos per enhancer element and determined the GFP expression patterns. Presence of an RFP signal in muscles of injected embryos served as control for presence of the ZED plasmid. PCR primers with attB sites used to amplify enhancer sequences are listed in S1 Table.

## Results

### *tcf12* expression patterns during early stages of development

To determine the spatiotemporal expression of *tcf12* throughout zebrafish development and its expression during cranial vault formation in adult stages, we generated transgenic *tcf12*:*EGFP* zebrafish, expressing an enhanced green fluorescent protein (EGFP) under control of the *tcf12* promoter fragment. By microinjection, we introduced the *tcf12*:*EGFP* plasmid and a Tol2 mRNA encoding the Tol2 transposase into fertilized zebrafish eggs [31]. By raising and outcrossing positively injected fish over subsequent generations we obtained a stable transgenic line with specific EGFP expression patterns driven by a 2.1 kb *tcf12* upstream promoter fragment, named *Tg(-2*.*1tcf12*:*EGFP)*.

**Fig 1:**
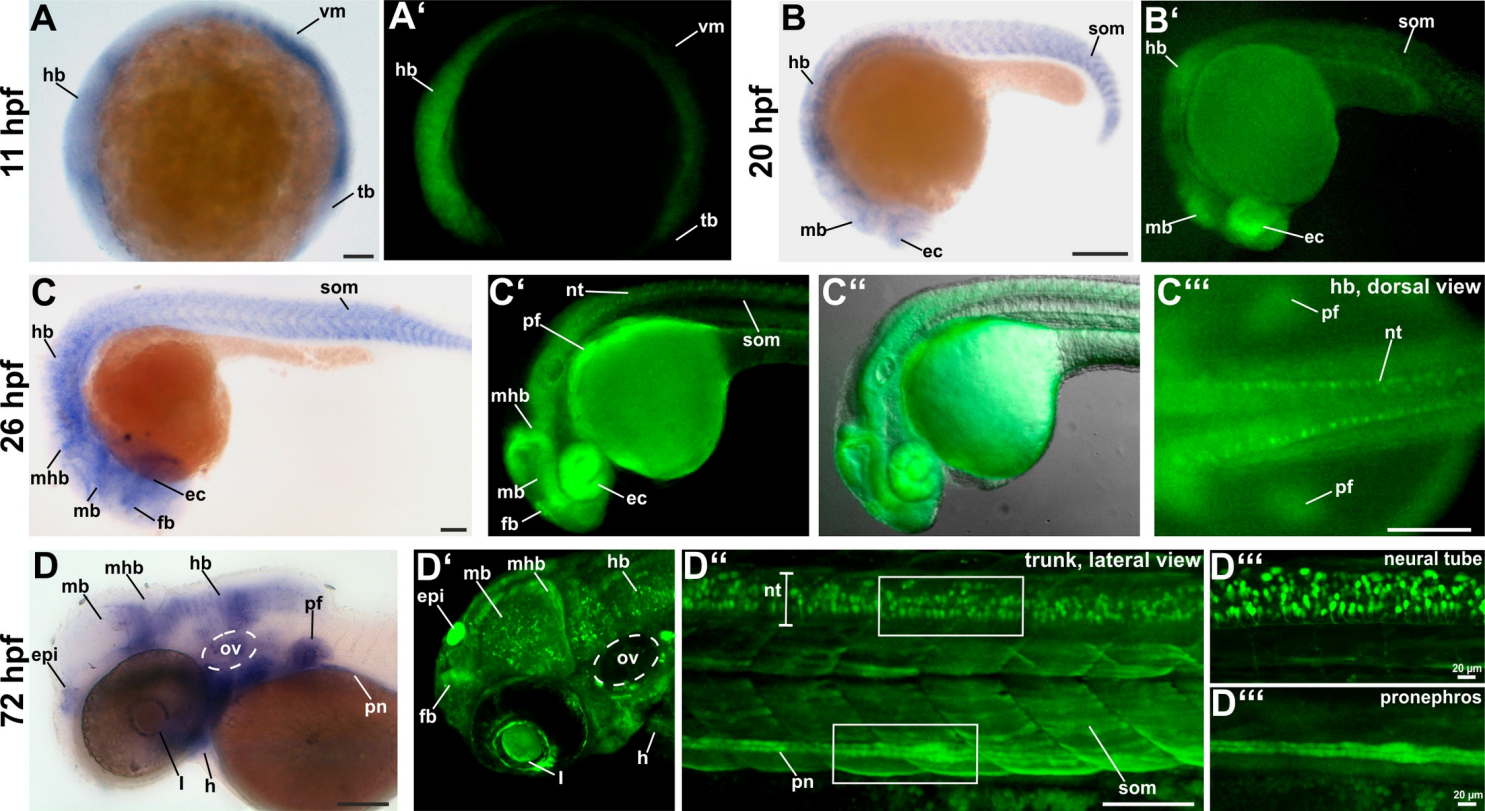
Comparison between endogenous *tcf12* mRNA expression detected by whole mount *in-situ* hybridization and EGFP expression in *Tg(-2*.*1tcf12*:*EGFP)* fish. (A, A’) *tcf12* transcripts are detectable by whole mount *in-situ* RNA hybridization at 11 hpf onwards in the hindbrain, ventral mesoderm, and tailbud. The expression pattern equals the first detectable EGFP signal in *Tg(-2*.*1tcf12*:*EGFP)* zebrafish. (B, B’) *In-situ* hybridization and transgenic embryos demonstrate *tcf12* expression in the eyecups, midbrain, hindbrain, and somites at 20 hpf. (C) 26 hpf old zebrafish embryos show an additional *tcf12* expression domain in the forebrain and the midbrain-hindbrain boundary. (C’, C”, C”‘) EGFP is also detected in the pectoral fin buds and in the neural tube of transgenic fish. (D, D’, D”; D”‘) In 72 hpf old fish *tcf12* expression can, in addition, be detected in the epiphysis, the otic vesicle, the pronephros, and the heart. Images in D”‘ show magnifications of the neural tube and pronephros shown in D”. ec, eye cups; epi, epiphysis; fb, forebrain; h, heart; hb, hindbrain; hpf, hours post fertilization; l, lens; mb, midbrain; mhb, midbrain-hindbrain boundary; nt, neural tube; ov, otic vesicle; pf, pectoral fins; pn, pronephros; som, somites; tb, tailbud; vm, ventral mesoderm. All scale bars represent 100 μm unless otherwise stated.

To validate if the fluorophore expression of the *Tg(-2*.*1tcf12*:*EGFP)* fish line resembles endogenous *tcf12* mRNA, we compared the fluorescence patterns of *tcf12* promoter driven EGFP expression to the expression patterns detected by *tcf12* whole mount *in-situ* hybridization at different embryonic stages (Fig 1; a list of all detected *tcf12* transcripts can be found in S2 Table). Early zebrafish *tcf12* expression was clearly detectable at 11 hpf (3 somite stage) in the hindbrain, the ventral mesoderm and the tailbud by *in-situ* hybridization and in the transgenic individuals (Fig 1A and 1A’). At 20 hpf, whole mount *in-situ* hybridization and the transgenic zebrafish revealed *tcf12* expression in the eye cups, the midbrain, the hindbrain and in the somites (Fig 1B and 1B’). Beyond that, *tcf12* is evidently expressed in the forebrain, midbrain-hindbrain boundary at 26 hpf and also in the pectoral fins and neural tube in transgenic fish (Fig 1C–1C”‘). From 72 hpf onwards additional expression in the epiphysis, the otic vesicle, the heart, pronephros, and eye lens was eminent in transgenic and non-transgenic fish (Fig 1D–1D”‘). Notably, the transgenic line showed localized, strong EGFP expression in single neurons of the central nervous system, e.g. in neurons of the midbrain, the midbrain-hindbrain boundary, the hindbrain, and the neural tube. These findings indicate that the *tcf12*:*EGFP* transgenic zebrafish show equivalent expression patterns to the endogenous *tcf12* mRNA expression determined via *in-situ* hybridization and allow high resolution analyses during development.

### Different *tcf12* promoter fragments reveal different gene expression patterns in transgenic zebrafish

**Fig 2:**
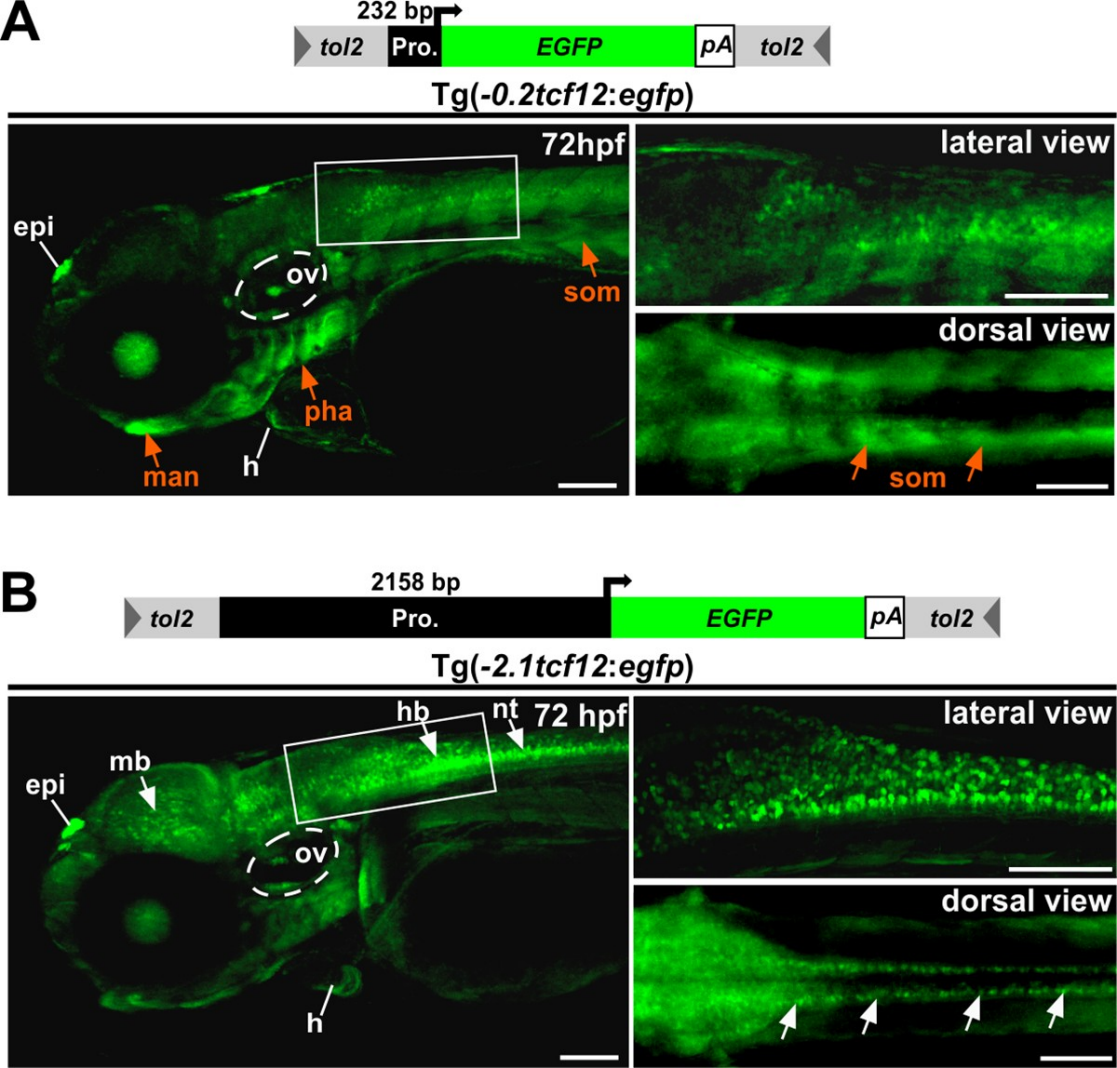
Comparison of EGFP expression in *Tg(-0*.*2tcf12*:*EGFP)* and *Tg(-2*.*1tcf12*:*EGFP)* fish. Comparison between different *tcf12*:*EGFP* transgenic lines. (A) The minimal promoter driven *Tg(-0*.*2tcf12*:*EGFP)* individuals show strong EGFP expressions in the gills (Pharyngeal arches), the lower jaw (mandible) and the somites (orange arrows). These expression domains are absent or rather weak in *Tg(-2*.*1tcf12*:*EGFP)* fish (B). *Tg(-2*.*1tcf12*:*EGFP)* zebrafish show a strong EGFP signal in neurons of the mid- and hindbrain and the neural tube (white arrows in B), which are lacking in the *Tg(-0*.*2tcf12*:*EGFP)* animals. An EGFP expression in the heart, the otic vesicle, and the epiphysis is detectable in both transgenic lines. epi, epiphysis; h, heart; hb, hindbrain; hpf, hours post fertilization; man, mandible; mb, midbrain; nt, neural tube; ov, otic vesicle; pha, pharyngeal arches; som, somites. All scale bars represent 100 μm unless otherwise stated.

To test whether a smaller “core promoter” sequence is likewise sufficient to mimic the spatiotemporal expression pattern of *Tg(-2*.*1tcf12*:*EGFP)* fish, we deleted 1926 bp of the 2158 bp promoter contained in the original *tcf12*:*EGFP* construct, leaving only a 232 bp sequence directly upstream of the *tcf12* promoter (Fig 2A). Transgenic fish arising from this construct are henceforth referred to as *Tg(-0*.*2tcf12*:*EGFP)*. A direct comparison between the two transgenic *tcf12*:*EGFP* lines reveals similar expression patterns from early stages on, although some striking differences were eminent (Fig 2). Whereas *Tg(-2*.*1tcf12*:*EGFP)* zebrafish show a notably strong EGFP expression in a large number of neurons throughout all parts of the CNS, *Tg(-0*.*2tcf12*:*EGFP)* individuals only show scattered EGFP-positive neurons in the hindbrain. In contrast, *Tg(-0*.*2tcf12*:*EGFP)* transgenic fish show strong EGFP expression in the gills, the lens, the lower jaw, and the somites, which is absent or rather weak in *Tg(-2*.*1tcf12*:*EGFP)* fish (Fig 2). Tissues with comparable EGFP expression include the epiphysis, the heart, and the otic vesicle, and indicate shared regulatory sequences present in both promoter fragments i.e. the 232 bp fragment. Moreover, these findings indicate localization of specific regulatory binding sites in the longer fragment for transcription factors that control *tcf12* expression in different neuronal tissues and hint to a complex *cis*-regulatory network controlling *tcf12* expression.

### Investigation of *tcf12* expression during juvenile and adult stages of development

**Fig 3:**
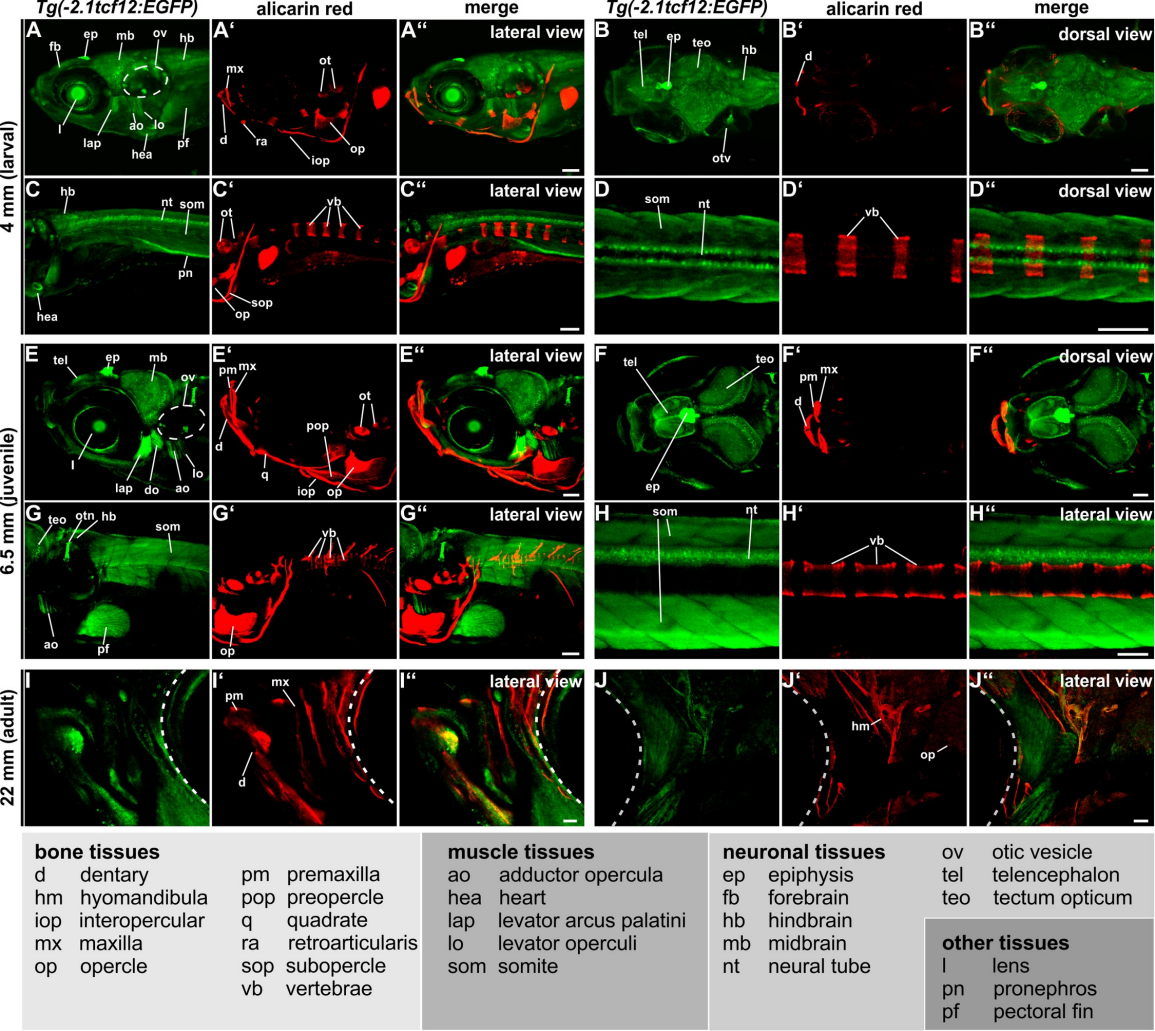
*Tg(-2*.*1tcf12*:*EGFP)* spatiotemporal EGFP expression during juvenile and adult stages. Investigation of EGFP expression over time *in vivo* in 4 mm (larval, 11 dpf), 6.5 mm (juvenile, 27 dpf) and 18 mm (adult, 90 dpf) old *Tg(-2*.*1tcf12*:*EGFP*) individuals via confocal imaging. Skeletal structures were stained with alizarin red. The *tcf12* expression profile can be split into three main tissue groups: bone, muscular, and neuronal. Besides these tissues, expression in the lens, pronephros, and pectoral fins is detected. Abbreviations can be found in the legend. All scale bars represent 100 μm. dpf, days post fertilization.

By regularly examining the transgenic reporter lines we were able to follow *tcf12* expression pattern from larval stages up to adult fish (Fig 3). For further investigations, *Tg(-2*.*1tcf12*:*EGFP)* individuals were preferred, as this line displayed a rather complete and specific expression pattern during early stages of development when compared to the mRNA expression (Fig 1). The observed EGFP expression during juvenile and adult stages can be subdivided into neuronal, bone and muscular tissues.

The neuronal gene expression pattern of the *Tg(-2*.*1tcf12*:*EGFP)* line enclose CNS neurons of the forebrain, including cells in the telencephalon and the epiphysis, the midbrain, including the optic tectum, and the hindbrain (Fig 3A–3C” and 3E–3G”). Moreover, EGFP is continuously detected in different neurons of the neural tube from 26 hpf onwards up to adulthood (Figs 1C’-1D”’, 3C–3D” and 3H–3H”). The most prominent EGFP signal in the transgenic fish is detectable in the epiphysis (also called pineal gland), a small endocrine gland which is involved in light detection and functions as circadian clock pace maker (Fig 3A–3B”and 3E–3F”) [36, 37]. Moreover, several neurons of the sensory organs show *tcf12*:*EGFP* expression, prominently observed in the otic vesicle and the eyes (Fig 3A–3B” and 3E–3F”).

Whereas in embryonic stages *tcf12* expression is mainly detected in neuronal tissues, juvenile and adult stages show strong EGFP expression also in different skeletal muscles. Besides the heart/myocard and the somites, high levels of muscular expression are detected in the viscerocranium (levator arcus palatini, levator operculi, adductor opercula, dilator operculi) of juvenile fish (Fig 3E–3E”).

Moreover, prominent EGFP expression is detected in a number of bones within the skull, e.g. the upper jaw (maxilla) and in lower jaw bones (dentary, interopercule) of juvenile *Tg(-2*.*1tcf12*:*EGFP)* fish (Fig 3E–3F” and 3G–3G”). In adult transgenic zebrafish additional EGFP expression in the hyomandibular and the opercle is visible (Fig 3J–3J”). Although the neural tube and somites show a strong *tcf12* promoter activity, no EGFP signal was detectable in the vertebrae (Fig 3C–3D”and 3G–3H”).

### Investigation of *tcf12* expression during suture development

To thoroughly assess the growth of calvarial plates during skull development and thereby establish a basis for future experiments we investigated untreated individuals of the *mitfa*^*w2/w2*^; *mpv17*^*b18/b18*^ strain, which are lacking melanophores and iridophores similar to *casper* mutants [38] over time from 15–36 mm (this corresponds to an age of 50–280 dpf) (S1 Fig). Visualization of calcified structures was routinely performed *in vivo* by alizarin red staining and subsequent confocal imaging. The *mitfa*^*w2/w2*^; *mpv17*^*b18/b18*^ fish allow easy observation of the growth phases of the calvaria plates *in vivo*. Before an age of 40 dpf (overall size <15 mm) all calvarial plates are separated and show no overlapping regions. Between 40 and 50 dpf (11 mm-15 mm) calvarial plate growth progressed quickly and resulted in convergence of the anterior and posterior skull plates forming the interfrontal and coronal sutures. The parietal bones are the last that overlap, forming the sagittal suture between 50 and 60 dpf (≥15 mm). Adult fish (>90 dpf, overall size ≥18 mm) possess completely developed cranial sutures (S1 Fig). In contrast to humans and mice, in zebrafish, all cranial sutures remain open throughout life [15]. Our results were in accordance to previously published data describing cranial suture patterning in zebrafish [18].

**Fig 4:**
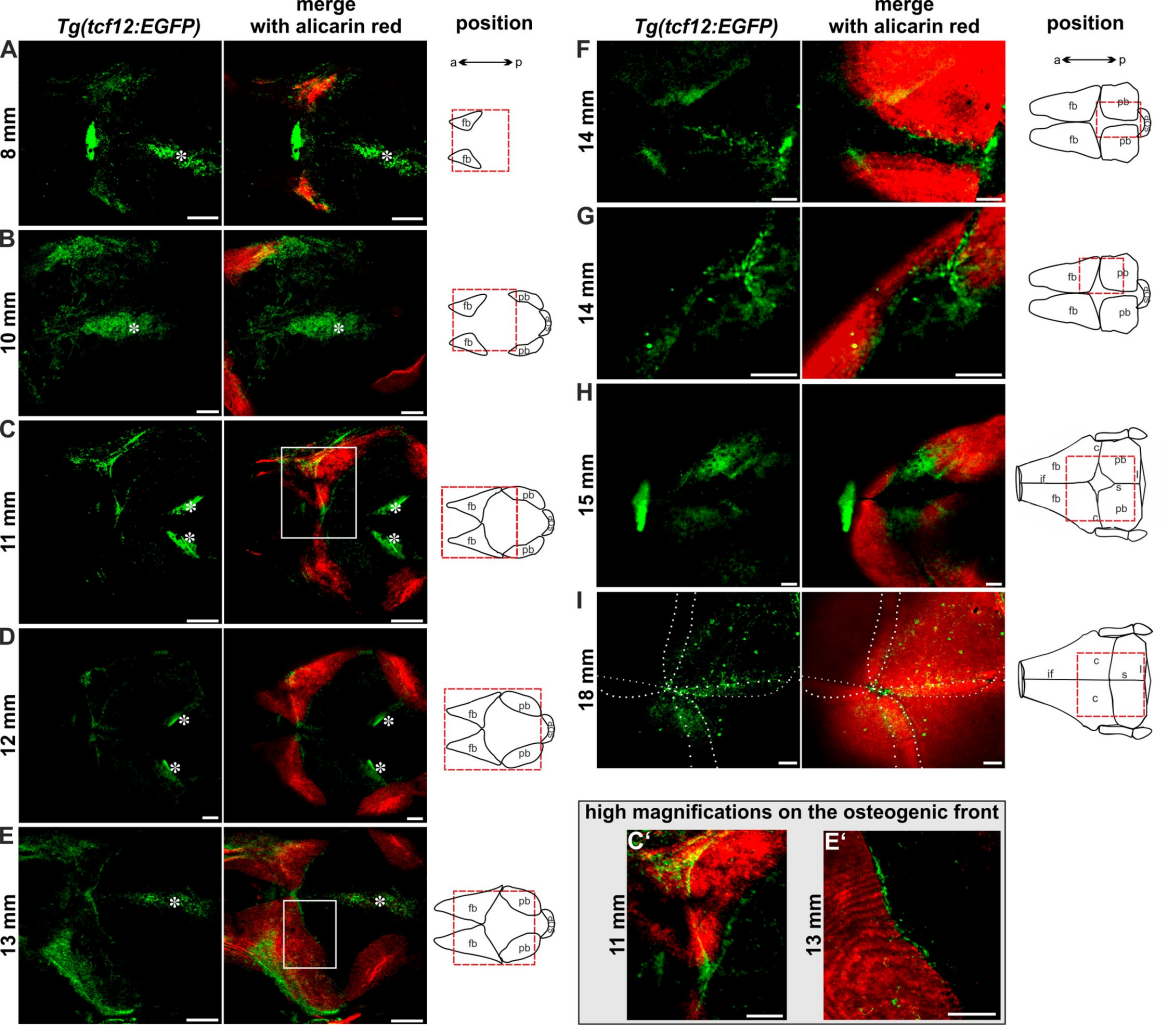
Expression of EGFP in *tcf12*:*EGFP* reporter lines during growth of calvarial bones and cranial suture patterning. Dorsal views of 8–18 mm (30–90 dpf) zebrafish skull vaults are depicted. Mineralized structures were stained with alizarin red. The schemes illustrate the growth stage of the calvarial bones. Red squares enclose the regions of the calvaria that are shown on the confocal images. EGFP signals in the center of the developing skull plates in A-E derive from the overlaying skin (marked with asterisk). C’ and E’ show detail magnifications of the boxed regions in C and E. The images display maximum intensity Z-projections from confocal stacks. Dashed white lines in I indicate fronts of the overlapping frontal and parietal bones. a, anterior; c, coronal suture; fb, frontal bone; if, interfrontal suture; l, lambdoid suture; p, posterior; pb, parietal bone; s, sagittal suture; sop, supraoccipital. All scale bars represent 100 μm.

After establishing imaging protocols and observation time frames, we examined EGFP expression in the *tcf12*:*EGFP* transgenic lines during suture development over time in fish of 8–18 mm in size (30–90 dpf; Fig 4). A striking observation was that both transgenic lines (*Tg(-0*.*2tcf12*:*EGFP)* and *Tg(-2*.*1tcf12*:*EGFP)*) mark *tcf12*:*EGFP* positive cells in the developing skull vault in a similar manner, indicating that the small 232 bp promoter sequence is sufficient to drive *tcf12* expression during cranial vault development. At 8 mm size the frontal skull plates of zebrafish are the first that begin to form, originating from single ossification centers. From these centers, ossification proceeds radially towards the bone periphery. EGFP positive cells are detected particularly at the ossifying frontal bones (Fig 4A). Additional EGFP positive cells that are spread through the center of the calvaria, relate to the overlaying skin (marked with asterisk in Fig 4A). At a size of 10 mm the EGFP signal shifts to the highly proliferative posterior zones of the frontal bones, recognizable by lighter red staining (Fig 4B). EGFP expression is also detected in the mesenchymal cells between the frontal bones. At this stage, the ossification centers of the parietal plates take shape, showing a slower ossification compared to the frontal bones, lacking EGFP signal. By 11 mm, ossification of the frontal bones proceeds quickly and EGFP positive cells can be detected surrounding the ossifying edges of the frontal bones (Fig 4C). At this point in time, the edges of the calvarial plates firstly converge forming the interfrontal and coronal sutures. EGFP positive cells are present in the areas where the interfrontal suture begins to form. Remarkably, at the anterior and posterior growth fronts of the frontal bones, EGFP positive cells align in a clearly visible row at a growth area, were unmineralized bone matrix and differentiated osteoblasts are located (Fig 4C and 4C’). This distinctive expression profile indicates that *tcf12* is involved in the regulation of osteoblast differentiation. Shortly thereafter, at a size of 12 mm, *tcf12*:*EGFP* positive cells also mark the evolving coronal suture and are visible around the growing parietal bones (Fig 4D). At a size of 13 mm, the frontal bones are still increasing in size rapidly. At this stage, EGFP is still detectable at the growth fronts of the frontal bones (Fig 4E and 4E’), with a notable signal in the area, where the frontal bones first meet, forming the interfrontal suture and where the frontal bones meet the parietal bones, forming the coronal sutures (Fig 4E). Once more, a strong EGFP signal is detectable in the center of the calvaria, together with an RFP signal, arising from pigment cells of the skin lying above the developing calvaria (Fig 4E asterisks). By 14 mm the parietal bones grow rapidly and the sagittal suture becomes apparent with EGFP positive cells accumulating inside the emerging suture and at the ossification zones of the parietal plates (Fig 4F). The lateral part of the coronal sutures is now formed. At this time point *tcf12*:*EGFP* cells are clearly apparent in the coronal sutures and again at the growth fronts of the frontal and parietal bones where the coronal and sagittal sutures form (Fig 4G). By 15 mm, an especially strong EGFP signal is detected at the posterior part of the frontal bones, the anterior part of the parietal bones and in the coronal suture mesenchyme, leaving out the area of the fontanel (Fig 4H). In adult zebrafish with a size of ≥18 mm, the frontal and parietal bone plates overlap, and all cranial sutures are fully developed. At this stage, the *tcf12*:*EGFP* lines mark cells on top of the calvarial bones and within the coronal and sagittal sutures. A weaker EGFP signal can be detected in the interfrontal suture (Fig 4I).

**Fig 5:**
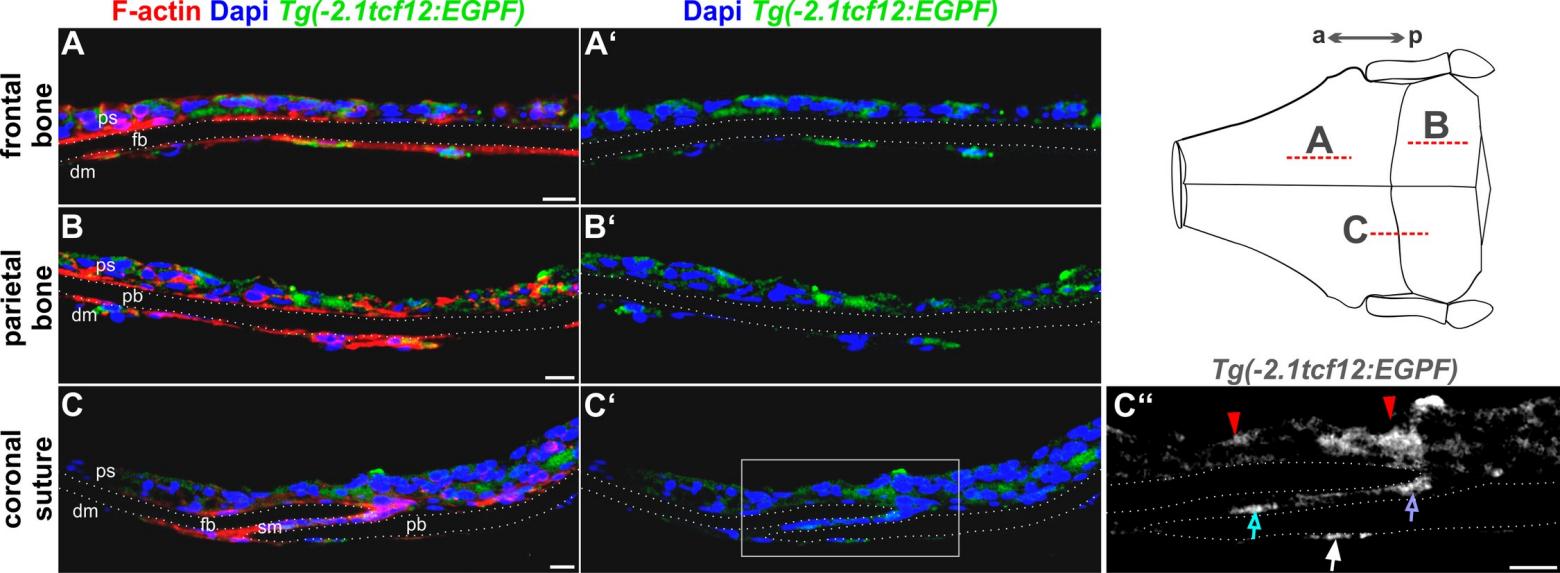
Cryosections of the calvaria reveal precise localization of *tcf12*:EGFP-expressing cells. Confocal images of immunostaining on cryosections of the frontal and parietal bones and the coronal sutures of adult *Tg(-2*.*1tcf12*:*EGFP)* fish. Specimens were stained with anti-GFP (green) and counterstained with Phalloidin (F-actin, red) and DAPI (blue) for visualization of cell structures and nuclei. Sections of the frontal (A) and parietal (B) bones reveal *tcf12*:*EGFP* expressing cells in the periosteum and dura mater. (C) Section of the coronal suture unveils a GFP expression in the periosteum (red arrows in C”) and dura mater, too (white arrow in C”). A detail magnification (C”) unveils an additional GFP expression in the suture mesenchyme of the coronal suture (blue arrow in C”) and at the tip of the osteogenic front of the frontal plate (purple arrow in C”).The dashed lines in the scheme mark the area of the cryosections shown in A-C”. a, anterior; dm, dura mater; fb, frontal bone; p, posterior; ps, periosteum; pb, parietal bone; sm, suture mesenchyme. All scale bars represent 10 μm.

To further clarify *tcf12* expression at a cellular level within the sutures we investigated the EGFP signal in the transgenic fish in greater detail by use of immunohistochemical staining with an anti-GFP antibody on cryosections of the skull of adult *Tg(-2*.*1tcf12*:*EGFP)* individuals (Fig 5C). To determine the overall expression pattern in adult zebrafish skull bones, we additionally investigated the GFP expression in sections of the frontal and parietal bones (Fig 5A–5B’) and of the coronal suture, which is most frequently affected in patients with *TCF12* mutations (Fig 5C–5C”). In all skull sections, a strong GFP signal is detected in the dermis which overlays the skull bones. In addition, single GFP positive cells of the dura mater, directly underlying the skull bones, were clearly detected (Fig 5A–5C’). A closer look reveals GFP positive cells inside the suture mesenchyme of the coronal suture and at the tips of the osteogenic fronts of the calvaria, presumably constituting osteoprogenitor cells or differentiated osteoblasts (Fig 5C”).

### Functional testing of three potential *tcf12* enhancer elements

**Fig 6:**
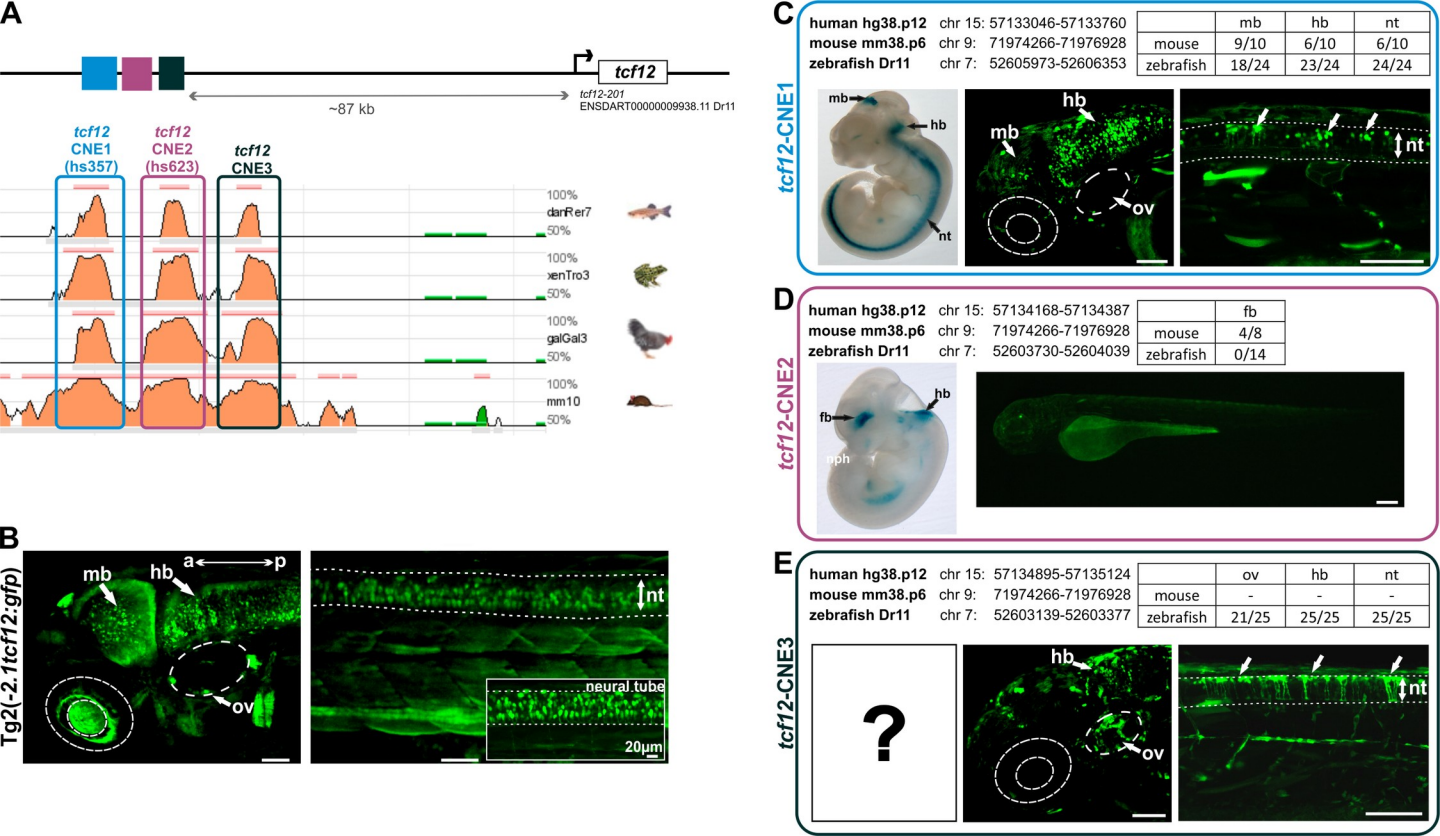
*In vivo* studies of evolutionarily conserved non-coding elements (CNEs) near the *tcf12* locus identified two enhancer elements that drive specific neuronal expression. (A) Graphic representation of the location of *tcf12*-CNE1-3 located ~87 kb 5’ of the *tcf12* gene (transcript tcf12-201 ENSDART00000009938.11) and interspecies alignments using the ECR browser [39]. (B) Fluorescent images of head and trunk of the *Tg(-2*.*1tcf12*:*EGFP)* line. (C to E) Comparison of the expression pattern of whole-mount lacZ staining of transgenic mice embryos from the VISTA Enhancer Browser [22] with fluorescent images of transient transgenic zebrafish embryos expressing *tcf12*-CNE1, *tcf12*-CNE2 or *tcf12*-CNE3. Genomic locations of the CNEs in human, mouse, and zebrafish are indicated. The table summarizes the number of mice and fish analyzed with the number of different expression pattern detected. Dotted lines mark the boundaries of the neural tube. a, anterior; hb hindbrain; mb, midbrain; nt, neural tube; ov, otic vesicle; p, poserior. All scale bars represent 50 μm unless otherwise stated.

After determining the exact expression pattern of *tcf12* with help of the established transgenic fish lines we attempt to analyze and verify potential upstream *tcf12* enhancer elements. To search for potential enhancer elements, we used the VISTA enhancer browser [22]. Two highly conserved noncoding human elements (Hs357: 714 bp and Hs623: 219 bp) located within the *TCF12* gene region were shown to display distinct enhancer activity in transgenic mice at 11.5 days post-coitum (referred to as *tcf12*-CNE1 for Hs357 & *tcf12*-CNE2 for Hs623). By use of the ECR browser [39], we identified an additional CNE (referred to as *tcf12*-CNE3) in the immediate vicinity of the other two CNE elements, showing high sequence conservation between human, mouse, frog, chicken, and zebrafish. The three CNEs are directly adjacent to one another, located in a genetic cluster about 87 kb 5’ of the *tcf12* gene (transcript *tcf12*-201 ENSDART00000009938.11) (Fig 6A). To test if the three CNEs function independently as enhancers in zebrafish, we cloned them into the zebrafish Enhancer Detector vector [26] and injected them into one-cell embryos. The injected embryos were observed until 3 dpf and transient EGFP expression was evaluated and compared.

Examination of *tcf12*-CNE1 revealed *EGFP* expression in the midbrain region in 9/10 tested mice (Fig 6C). 6/10 murine embryos showed expression in the hindbrain and the neural tube. Our *in vivo* studies for *tcf12*-CNE1 unveiled that 18/24 of the examined zebrafish exhibit a distinct EGFP expression in the midbrain, 23/24 in the hindbrain and all investigated fish (24/24) showed EGFP-expressing neurons within the neural tube. The examined EGFP expression in the hindbrain region of transgenic *tcf12*-CNE1:*EGFP* fish was particularly striking since a high number of EGFP expressing neurons was detectable in the posterior part of the hindbrain (Fig 6C). This observation contrasts starkly with the EGFP expression of the *Tg(-2*.*1tcf12*:*EGFP)* line, that showed higher EGFP expression in neurons localized in the anterior part of the hindbrain (Fig 6B). Analysis of the *tcf12*-CNE1:*EGFP* fish also detected EGFP-positive neurons dorsal of the neural tube, that are not EGFP-positive in the *Tg(tcf12*:*EGFP)* line (Fig 6B and 6C). Similar neuronal EGFP expression was detected in the anterior part of the hindbrain and in neurons of the midbrain in both, *Tg(tcf12-CNE1*:*EGFP)* fish and *Tg(-2*.*1tcf12*:*EGFP)* fish. These findings lead to the conclusion that *tcf12*-CNE1 is a specific enhancer element for neurons of the mid- and hindbrain and the neural tube, highly conserved between human, mouse, and zebrafish.

In mice, *tcf12*-CNE2 showed expression in neurons of the forebrain in 50% (4/8) of the analyzed mice (Fig 6D). Interestingly, recent studies in mice showed, that deletion of CNE2 (hs623) causes a reduction of *Tcf12* expression in the sub-ventricular zone, which is located along the lateral ventricles, harboring neural stem cells [40]. Our functional testing in zebrafish revealed no EGFP expression in any of the investigated *tcf12*-CNE2:*EGFP* zebrafish (0/14), indicating that *tcf12*-CNE2 does not operate as enhancer element in zebrafish (Fig 6D).

The evaluation of *tcf12*-CNE3 revealed strong EGFP expression in the hindbrain and neural tube in all examined zebrafish embryos (25/25) and a remarkable expression in clusters of neurons surrounding the otic vesicle in 84% of the analyzed fish (21/25), (Fig 6E). The expression pattern in the otic vesicle and neural tube is not detectable in the *Tg(tcf12*:*EGFP)* individuals, strongly indicating a specific function of *tcf12*-CNE3 in neurons of these tissues (Fig 6B and 6E). In mice, *tcf12*-CNE3 has not been analyzed yet.

## Discussion

In this study, we present the establishment of a novel transgenic model for observing the changing expression pattern of *tcf12* during zebrafish development and cranial suture morphogenesis. In addition, we tested the transcriptional activity of three non-coding elements within the *tcf12* locus and compared their activity to the transgene expression of *tcf12*:*EGFP* fish.

### Newly established *tcf12*:*EGFP* reporter lines reproduce endogenous mRNA expression

By use of the Tol2 system, we generated a stable transgenic *Tg(-2*.*1tcf12*:*EGFP)* reporter line, expressing EGFP under control of a 2158 bp upstream sequence of several *tcf12* transcripts, incorporating the transcript coding for the *tcf12* reference sequence (NCBI Reference Sequences: NM_214816.1 and NP_999981.1).

Via comparison to *in-situ* hybridization, we were able to verify the specificity of the *tcf12* expression patterns displayed in the transgenic zebrafish embryos. By reducing the promoter sequence to 232 bp, we additionally generated a second stable transgenic line *Tg(-0*.*2tcf12*:*EGFP)*, demonstrating that this short upstream sequence is sufficient to drive *tcf12* gene expression activity. This observation corresponds to the expectations that the short 232 bp fragment represents the minimal proximal promoter element that incorporates the core promoter containing an RNA polymerase binding site, a TATA box, and a transcription start site.

A comparison between the two transgenic lines indicated overlapping EGFP expression patterns in a number of tissues, including the epiphysis and the heart. Remarkably, the *Tg(-0*.*2tcf12*:*EGFP)* line marks a significantly smaller amount of EGFP-positive neurons in all investigated CNS regions and shows a broader expression in the somites, the gills and the lower jaw of the zebrafish. The *Tg(-2*.*1tcf12*:*EGFP)* fish closely resemble the neuronal expression pattern of *tcf12* mRNA expression as detected via *in-situ* hybridization and thereby hint to additional regulatory elements in this distal promoter element. Further comparison to published *tcf12* expression data in the ZFIN expression database by Thisse et al. confirmed that the observed *in-situ* mRNA and the transgene EGFP expression presented in this study resembles a reproductive pattern [41–43]. Especially *tcf12* expression in neuronal tissues, the pectoral fins, and the branchial arches which form the gills was independently detected in all experiments. Differences could be observed in a rather low *EGFP* expression during somitogenesis (16–26 hpf) and in a strong *EGFP* expression in the pronephros of the transgenic individuals (72 hpf).

Our combined *in vivo* and *in-situ* observations enable us to follow *tcf12* expression over a wide time range. Table 1 sums up the commonalities and differences in gene expression in different tissues from embryonic stages up to adulthood.

**Table 1:** Summary of *tcf12* expression in zebrafish throughout development. List of expression patterns detected in *tcf12*:*EGFP* transgenic embryos, juvenile and adult zebrafish. Expression profiles are divided into neuronal, muscle, bone and other tissues.

	embryo	juvenile	adult
**neuronal tissues**	fore-, mid-, and hindbrain	fore-, mid-, and hindbrain	fore-, mid-, and hindbrain
	epiphysis	epiphysis	epiphysis
	spinal cord neurons	spinal cord neurons	spinal cord neurons
	otic vesicle neurons	otic vesicle neurons	otic vesicle neurons
**muscule tissues**	somites/muscle precursors	somites	somites
	heart	heart	heart
		muscles of the viscerocranium	muscles of the viscerocranium
**bone tissues**	precursors of jawbones	bones of the viscerocranium	bones of the viscerocranium
			cranial bones
**other tissues**	pharyngeal arches		
	eye/ lens	lens	lens
	pronephros	pronephros	pronephros
	pectoral fins	pectoral fins	pectoral fins
			cranial sutures

### *tcf12* expression patterns in neuronal and muscular tissues resemble areas of *TCF12* function in higher vertebrates

With the newly established transgenic fish lines, a fine-grained expression pattern of *tcf12* is detectable right up to single EGFP-positive cells throughout development. The *Tg(-2*.*1tcf12*:*EGFP)* line reveals a rather broad expression pattern of *tcf12* from 11 hpf onwards throughout development. This observation fits the general expectation of a broad E-protein expression, also observed in other vertebrates [44, 45]. Functional and tissue specificity of *tcf12* is proven to be induced by heterodimer formation with other bHLH proteins, e.g. ID-proteins, and also depends on its own spatiotemporal expression pattern [46, 47]. TCF12 has been shown to directly bind to TWIST1 during bone development [7, 9], MYOD during muscle development [48, 49] and NeuroD2 during brain development [4].

With the established transgenic lines, we were able to unveil the tissue-specific *tcf12* expression levels and patterns. Our findings indicate a remarkable high expression in neurons of the midbrain, hindbrain and neural tube in *Tg(-2*.*1tcf12*:*EGFP)* fish. These findings are consistent with studies in mice and rats showing that *Tcf12* plays a key role in neuronal differentiation of the brain, the notochord and the neural tube [5, 6, 50–53]. Beyond that, knockout studies in mice showed, that besides severe defects of B- and T-cell development, *Tcf12* null mice and *Tcf12*^*dm*^ homozygous mice develop an exencephaly of low penetrance and show a high percentage of postnatal death [3, 54, 55].

Besides neuronal expression patterns, the transgenic fish also revealed strong EGFP expression in muscular tissues, like the somites, muscles of the viscerocranium (adductor opercula, levator muscles) and in the heart myocard. Parker et al. showed a similar *TCF12* expression pattern in mice regulating the proliferation and differentiation of myoblasts [48], while Hu et al. found *TCF12* to be highly expressed in skeletal muscle tissue of humans [47]. Using the transgenic lines, muscle-specific *TCF12* functions and its interactions with muscular transcription factors can be investigated in the future.

### Investigation of *tcf12* expressing cells during bone and suture development enable spatiotemporal visualization of craniofacial structure formation

First expression of *tcf12*:*EGFP* in developing bony structures could be revealed in juvenile fish in bones of the jaw, representing the first formed bone structures during development [56, 57]. In the vertebrae, which ossify from ~7 dpf onwards [58], no EGFP signal was detectable throughout development. Adult transgenic individuals mark *tcf12* positive cells within the hyomandibular, the tooth buds, and the opercle. These findings further indicate that *tcf12* is an important factor to regulate gene expression in the zebrafish jaw and dentary.

Of special interest in our study was the investigation of *tcf12* expression during cranial suture development. Normal development of the cranial sutures underlies a complex regulatory network which includes the suture mesenchyme, the osteogenic fronts of the skull bones, the underlying cells of the dura mater, as well as the brain [59–61]. The complexity of the interplay of different tissues comes along with several interacting signaling pathways that are involved in cranial vault formation. Over the last years, a number of different mutations in *TCF12* have been identified to cause craniosynostosis in humans [9, 62, 63]. By use of cell culture experiments and *in vivo* studies in mice and zebrafish, researchers were able to show that *TCF12* plays an important role in osteogenic differentiation and in cranial bone modeling [7, 9, 21]. Studies in mice and zebrafish could further identify Twist1 as an important interaction partner of Tcf12 in cranial suture patterning since a loss of *Tcf12* caused coronal suture fusions only in combination with a *Twist1* loss [9, 21]. These data are in contrast to observations in human patients, as heterozygous mutations in *TCF12* alone are sufficient to cause craniosynostosis.

By visualizing the EGFP expression during cranial vault development in both transgenic lines, we were able to detect *tcf12* expression at the leading edges of the ossifying cranial bones throughout craniofacial development. This observation confirms the assumption of Yi et al. that *tcf12* plays an important role in the process of osteogenic differentiation in zebrafish [7]. A particularly striking observation was that transgenic fish of the *Tg(-0*.*2tcf12*:*EGFP)* line displayed EGFP expressing cells at the bone fronts and inside the overlapping sutures comparable to *Tg(-2*.*1tcf12*:*EGFP*) fish. This finding shows, that the 232 bp upstream sequence is sufficient to regulate the spatiotemporal *tcf12* expression in the calvaria of zebrafish. As soon as the sutures take shape, the transgenic lines highlight *tcf12* expressing mesenchyme cells not only within the developing coronal but also in interfrontal and sagittal sutures. Expression in all three suture types can also be detected in adult individuals. These findings are in accordance with Teng et al., who detected *tcf12* expression in skull plates of adult zebrafish via *in-situ* hybridization [21]. Humans, mice, and zebrafish show a selectively high sensitivity of fusions in the coronal sutures as a consequence of combined mutations within *tcf12* and *twist1* [8, 9, 21]. The underlying cause of this differential sensitivity could not be determined until now. Even though we could detect a *tcf12*:*EGFP* signal in all sutures, further studies are necessary to determine if different amounts of EGFP expressing cells can be detected in the different suture types and if these stochastic differences predispose to specific suture fusions. In addition, it is conceivable that similar to Twist1 [64], TCF12 shows a different dimer partner selection in the coronal sutures compared to the interfrontal and sagittal suture that is critical for the regulation and function of the coronal suture. Visualization of the spatiotemporal EGFP expression of the skullcaps of *tcf12*:*EGFP* individuals indicates that *tcf12* plays an important role in regulating the osteogenic differentiation. Additionally, *tcf12* expression appears to be upregulated in actively ossifying areas of the skull bones depending on the developmental time point and thus indicates different phases of *tcf12* expression during suture establishment.

By performing cryosections, we were able to reveal the exact expression profile of *tcf12* in the calvaria of adult transgenic fish. Our findings indicate that *tcf12* is expressed in the skin overlying the calvaria and in cells located immediately below the calvaria, presumably the dura mater. Several studies already reported that the dura mater plays an important role in suture patency and that secreted growth factors like FGF2, BMP4, and TGFβ1–3 are expressed from this tissue. As consequence, lack of the dura mater leads to suture fusion [60, 61, 65–67]. Moreover, our findings are consistent with recent research, showing that *Tcf12* is highly expressed in undifferentiated mesenchymal stem cells *in vitro* [7]. Interestingly, *tcf12*:*gfp* expression is also found at the tips of the osteogenic fronts in adult transgenic fish. In this region, lifelong osteogenic differentiation takes place and *tcf12*:*EGFP* expression might mark the corresponding bone stem cell pool necessary for this feature in teleost fish [18]. Altogether, our findings further indicate that *tcf12* is involved rather in craniofacial bone development than in general skeletal development.

### Functional testing of potential enhancer elements identifies two CNEs within the zebrafish *tcf12* locus

Besides general *tcf12* expression, we investigated the tissue-specific activity of potential regulatory elements located in an evolutionary conserved upstream locus. Genetic research has shown that mutations in regulatory elements of genes can result in severe developmental defects, thus making screens for regulatory mutations more and more important [68–74]. Over the last years, zebrafish have been shown to be a valuable model for *in vivo* detection and validation of human enhancer elements [24, 25, 75]. On the one hand, tools like the zebrafish enhancer detection (ZED) vector enable the establishment of transgenic zebrafish reporter lines fast and easily [26]. On the other hand, CNEs conserved between distant species like human and zebrafish are more likely to be functional than those among closely related species like human and mouse [76]. Understanding the regulation of the gene expression of genes like *tcf12*, that are involved in developmental defects like craniosynostosis, is therefore important for understanding the disease mechanism and to find a treatment for the patients in the long term.

By using the transgenic *tcf12*:*EGFP* fish as reference to compare three potential *tcf12* enhancer elements *in vivo* using the ZED vector [26], we could confirm two CNEs out of three as being specific *tcf12* enhancer elements driving EGFP expression mainly in neuronal regions of zebrafish embryos during the first days of development (*tcf12*-CNE1 and *tcf12*-CNE3). These elements are highly conserved in vertebrates and might resemble evolutionary conserved transcription factor binding sites acting upstream of *tcf12* expression. An explanation for the observation, that *tcf12*-CNE2 does not operate as enhancer element in zebrafish, is that there is a different need in zebrafish for other regulatory elements that are absent in this CNE sequence to regulate gene expression. Even though *tcf12*-CNE2 does not act as enhancer element in zebrafish, it can still be considered as potential *TCF12* enhancer in higher vertebrates like humans and mice.

Over the last years, mutations in enhancer elements, as well as structural aberrations affecting CNEs could be associated with various diseases, including skeletal disorders [69–71, 77]. Klopocki and colleagues, for example, have shown that copy-number variations involving the *IHH* locus are associated with syndactyly and craniosynostosis [78]. Studies in mice showed that deletion of an enhancer element of the *Hoxc8* gene leads to skeletal defects similar to those of *Hoxc8*^−/−^ mice [70]. Our analysis paves the way for future studies to address the question, whether the identified enhancer elements act on *tcf12* expression in other tissues, e.g. the cranial sutures by binding essential upstream factors of *tcf12* transcriptional regulation. In this regard, it would be of great interest to investigate if mutations in these non-coding elements can act as risk factors leading to craniosynostosis in human patients. Taking a closer look at the *cis*-regulatory elements of genes associated with congenital developmental defects like craniosynostosis will enable a better understanding of the regulation of these genes and may point to a new pathomechanism underlying yet unsolved craniosynostosis cases.

## Supporting information

S1 Fig Suture visualization in zebrafish. Alizarin red staining was performed at different stages of development (A: 15 mm, 50 dpf; B: 26 mm, 120 dpf; C: 36 mm, 280 dpf/adult) and showed calvarial plate growth and progression of suture establishment over time. Dorsal views are maximum intensity Z-projections from confocal stacks.
(TIF)

S1 Table Primer sequences. (DOCX)

S2 Table Detailed information about different zebrafish *tcf12* transcripts, in-situ hybridization targets and transcripts driven by the transgenic promoter fragments. (XLSX)
