# Supplementary figures and images for: On the traces of tcf12: Investigation of the gene expression pattern during development and cranial suture patterning in zebrafish (Danio rerio)
Source: PLoS One. 2019 Jun 12;14(6):e0218286. doi: 10.1371/journal.pone.0218286 (PMC6561585; doi:10.1371/journal.pone.0218286)

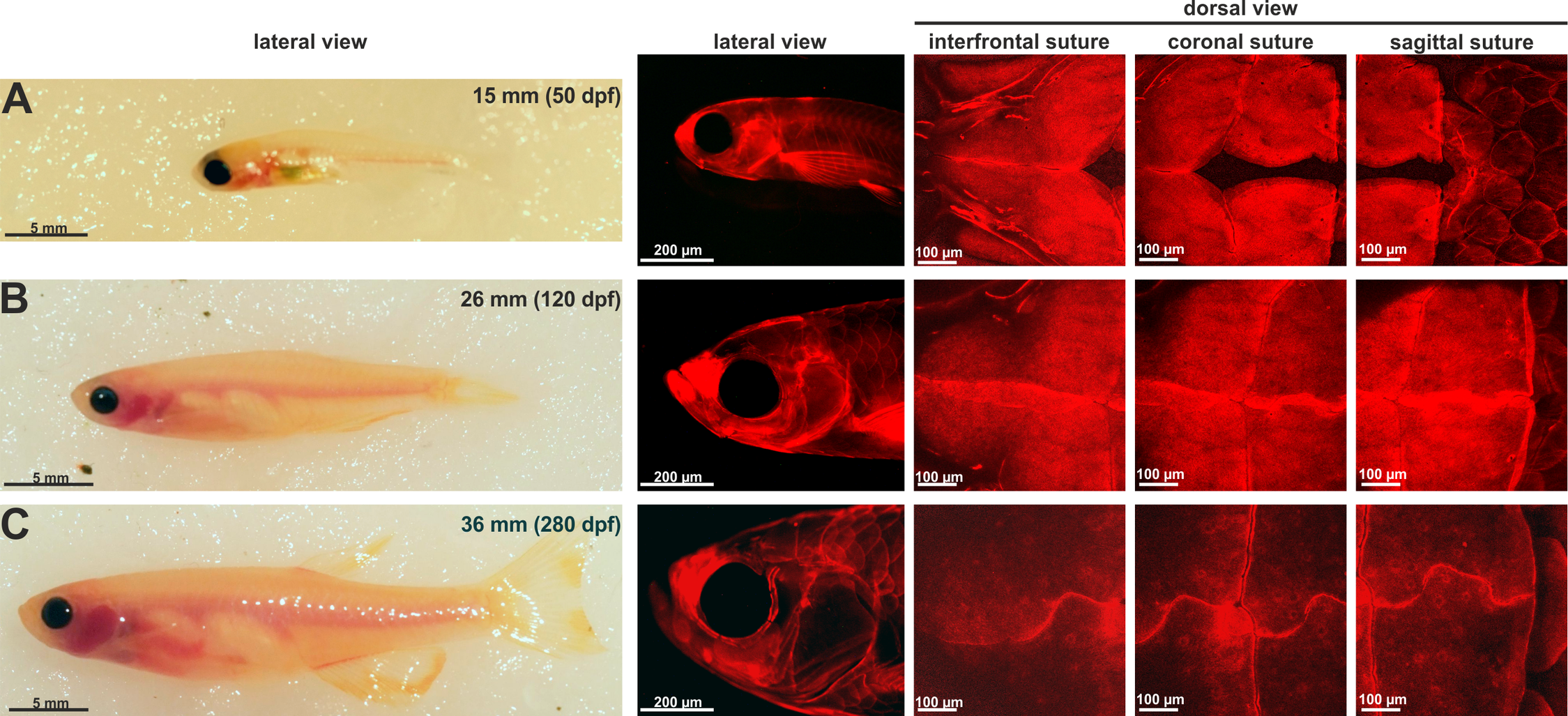

Supplement: S1 Fig — Alizarin red staining was performed at different stages of development (A: 15 mm, 50 dpf; B: 26 mm, 120 dpf; C: 36 mm, 280 dpf/adult) and showed calvarial plate growth and progression of suture establishment over time. Dorsal views are maximum intensity Z-projections from confocal stacks. (TIF) [file pone.0218286.s001.tif]
