# Supplementary material for: On the traces of tcf12: Investigation of the gene expression pattern during development and cranial suture patterning in zebrafish (Danio rerio)
Source: PLoS One. 2019 Jun 12;14(6):e0218286. doi: 10.1371/journal.pone.0218286 (PMC6561585; doi:10.1371/journal.pone.0218286)
Supplement: S1 Table — (DOCX) [file pone.0218286.s002.docx]

| **Cloning of *tcf12:EGFP* promoter constructs** | |
| --- | --- |
| *tcf12* promoter Forward *att*B4 | GGGGACAACTTTGTATAGAAAAGTTGCTCACTAGACTTCTTGCAGACA |
| *tcf12* promoter Reverse *att*B1R | GGGGACTGCTTTTTTGTACAAACTTGCACTTTTCACAAACCCGCTCC |
| **Cloning of *tcf12* enhancer elements** | |
| ZF *tcf12*-CNE1 attB Insert Forward | GGGGACAAGTTTGTACAAAAAAGCAGGCTTATGGCTCAGATGATGCGATATCCA |
| ZF *tcf12*-CNE1 attB Insert Reverse | GGGGACCACTTTGTACAAGAAAGCTGGGTTAGAATCTCACAGGTTTTTGCAGCAT |
| ZF *tcf12*-CNE2 attB Insert Forward | GGGGACAAGTTTGTACAAAAAAGCAGGCTTAACATATGTAATTTTGTAGATTTGTATTATTATTATTATTATTGCT |
| ZF *tcf12*-CNE2 attB Insert Reverse | GGGGACCACTTTGTACAAGAAAGCTGGGTTACACACACAAACATCACAATGCCAA |
| ZF *tcf12*-CNE3 attB Insert Forward | GGGGACAAGTTTGTACAAAAAAGCAGGCTTAATTAGTATCCACACTCCACAGCCT |
| ZF *tcf12*-CNE3 attB Insert Reverse | GGGGACCACTTTGTACAAGAAAGCTGGGTTCCCCAGTTTGGATGTTTGCG |
| **whole mount *in-situ* hybridization** | |
| *tcf12* isH Forward | AGGTGTATTGGCCAGTCAGG |
| *tcf12* isH Reverse | GGCACTTGCCAGTTTTCTTC |
